# Association Between Radiographic Haglund Morphology and MRI-Measured Achilles Tendon Rupture Location: A Retrospective Cross-Sectional Study

**DOI:** 10.3390/diagnostics16213592

**Published:** 2026-10-30

**Authors:** Adnan Arslan, İrfan Narin, Ferhat Danışman, Aytekin Dikici, Bayram Ersidar, Yunus Can Ünal

**Affiliations:** Department of Orthopaedics and Traumatology, University of Health Sciences, Van Training and Research Hospital, Süphan Mahallesi, Hava Yolu Kavşağı 1. Kilometre, Edremit, Van 65300, Türkiye

**Keywords:** Achilles tendon rupture, Haglund deformity, posterior calcaneal morphology, rupture location, magnetic resonance imaging, lateral ankle radiography

## Abstract

**Background/Objectives:** Achilles tendon ruptures usually occur proximal to the calcaneal insertion. Whether posterosuperior calcaneal prominence is associated with rupture location remains uncertain. We examined the association between radiographic Haglund morphology and MRI-measured rupture-to-insertion distance. **Methods:** In this retrospective cross-sectional study, adults treated for acute Achilles tendon rupture from January 2015 to December 2025 were assessed. Rupture distance was measured on sagittal MRI. Two blinded orthopaedic surgeons measured bump height and the bump–calcaneus ratio on standard lateral ankle radiographs; radiographic Haglund morphology required both thresholds (≥4.0 mm and ≥7.5%, respectively). We compared groups with the Mann–Whitney U test and bootstrap confidence intervals and used median regression adjusted for age, sex, sport-related mechanism, and comorbidity. **Results:** Of 292 screened patients, 106 were included; 28 (26.4%) met the radiographic criteria. The cohort included 27 women (25.5%) and had a median age of 41.5 years. Interobserver ICC(2,1) values were 0.9976–0.9994, with complete agreement in threshold-derived classification (κ = 1.000). Median rupture distance was 1.60 cm (IQR, 1.49–2.58) with Haglund morphology versus 4.85 cm (IQR, 3.51–5.55) without it. The unadjusted median difference was −3.25 cm (95% CI, −3.40 to −2.68; *p* < 0.001); the adjusted association was −2.89 cm (95% CI, −3.61 to −2.17; *p* < 0.001). **Conclusions:** Radiographic Haglund morphology was associated with more distal Achilles tendon rupture in this selected rupture cohort. The retrospective design and exclusion of 113 patients with incomplete records limit generalizability; the study does not establish causality or whether morphology increases rupture risk.

## 1. Introduction

Acute Achilles tendon rupture is increasingly encountered in sports medicine and orthopaedic practice, with population-based studies demonstrating rising incidence over recent decades [1,2,3]. It frequently occurs during sport in physically active middle-aged adults and is commonly associated with sudden loading of the gastrocnemius–soleus complex. Although tears may occur at different levels, most involve the midsubstance several centimeters proximal to the calcaneal insertion. Magnetic resonance imaging studies have localized many acute ruptures approximately 5–8 cm proximal to the distal calcaneal insertion, and other clinical imaging studies have reported a similar distribution [4,5]. Rupture level may influence tendon morphology, gap characteristics, and clinical management; therefore, localization may represent a clinically relevant feature rather than a purely descriptive finding [5,6].

Radiographic Haglund morphology describes a prominent posterosuperior calcaneus adjacent to the Achilles tendon insertion and should be distinguished from symptomatic Haglund syndrome [7,8,9]. The posterior calcaneal tuberosity comprises superior, middle, and inferior facets. The superior facet is related to the deep retrocalcaneal bursa, whereas the middle and inferior facets form the Achilles tendon insertional footprint [10]. Traditional angular and line-based radiographic parameters have shown variable diagnostic performance, prompting the development of more quantitative measurements. Bump height and the bump–calcaneus ratio have been proposed as reproducible measures, with thresholds of 4.0 mm and 7.5%, respectively, demonstrating diagnostic utility [11]. In this study, their combined use was treated as a prespecified operational definition rather than a universal diagnostic criterion. The close anatomical relationship between the posterosuperior calcaneus and the distal Achilles tendon provides a plausible basis for localized tendon pathology. Imaging and clinical studies have linked prominent posterior calcaneal morphology with Achilles impingement and insertional tendinopathy [12,13,14], while altered compressive and tensile strain patterns have been demonstrated in insertional Achilles tendinopathy [15]. These observations support biological plausibility but do not establish a causal mechanism for acute rupture.

Despite this anatomical and biomechanical rationale, the relationship between radiographic Haglund morphology and the location of Achilles tendon rupture remains insufficiently characterized. Published reports of rupture in association with Haglund deformity are scarce and largely limited to individual case reports [16,17,18]. Conversely, studies of Achilles tendon rupture location have focused mainly on the distribution of rupture sites or the relationship between rupture level and subsequent clinical outcomes [4,5,6]. To our knowledge, no clinical series has adequately evaluated the association between objectively defined radiographic Haglund morphology and MRI-measured rupture-to-calcaneal insertion distance.

The primary objective of this study was to determine whether radiographic Haglund morphology was associated with the anatomical location of Achilles tendon rupture. We hypothesized that patients meeting the prespecified radiographic criteria for Haglund deformity would have a shorter MRI-measured rupture-to-calcaneal insertion distance, corresponding to a more distal rupture location, than patients who did not meet these criteria. Because Achilles tendon rupture occurs in both athletic and non-athletic settings, sport-related injury mechanism was included as a prespecified covariate in the adjusted analysis.

## 2. Materials and Methods

### 2.1. Study Design and Setting

This single-center retrospective cross-sectional study included adult patients treated for Achilles tendon rupture at a tertiary referral center in Türkiye between 1 January 2015 and 31 December 2025. Patients aged 18 years or older of either sex were eligible, with no upper age limit. The study protocol was approved by the SBÜ Van EAH Non-Interventional Clinical Research Ethics Committee (approval No. GOKAEK/2026-06-07; 12 June 2026). The study was reported in accordance with the Strengthening the Reporting of Observational Studies in Epidemiology (STROBE) statement [19].

### 2.2. Patient Selection

All patients treated for acute Achilles tendon rupture during the study period were screened. Patients were excluded if they were younger than 18 years; if the injury was related to a traffic accident, an open wound and/or osteomyelitis, or multitrauma; or if they had a chronic or neglected rupture. Patients were also excluded when any component required for standardized assessment was unavailable or technically inadequate. These reasons included incomplete clinical records; absence of MRI, including patients evaluated only by ultrasonography; MRI quality insufficient for reliable rupture-to-insertion distance measurement; and absent or technically inadequate lateral radiographs required for calcaneal measurements. These categories were not mutually exclusive, and separate patient-level counts for each category were unavailable.

The patient selection process is presented in the study flow diagram (Figure 1).

Demographic and clinical data were extracted retrospectively from the medical records. Variables included age, sex, affected side, injury mechanism, rupture completeness (partial or total), treatment modality (surgical or nonoperative), and recorded comorbidities. Comorbidities comprised diabetes mellitus, chronic kidney disease, hypertension, osteoporosis, and gout. Because individual patients could have more than one condition, “any comorbidity” indicated the presence of at least one recorded condition.

### 2.3. Assessment of Achilles Tendon Rupture Location

The primary outcome was the distance between the Achilles tendon rupture and its calcaneal insertion. MRI examinations were obtained within 1–7 days after presentation, before treatment, using the standard ankle imaging position. The sagittal slice that most clearly demonstrated both the rupture and calcaneal insertion was selected. Using the PACS linear measurement tool, the rupture-to-calcaneal insertion distance was measured as a straight line along the longitudinal axis of the Achilles tendon, from the calcaneal insertion to the most distal identifiable point of tendon discontinuity, and recorded in centimeters [4].

### 2.4. Radiographic Assessment of Posterior Calcaneal Morphology

Posterior calcaneal morphology was evaluated on standard lateral ankle radiographs obtained before treatment. The radiographs were stored in Digital Imaging and Communications in Medicine (DICOM) format and reviewed using the institutional picture archiving and communication system (PACS).

Bump height and the bump–calcaneus ratio were measured according to the method described by Tang et al. [11]. A baseline tangent to the anterior tubercle and medial tuberosity of the calcaneus was first drawn along the inferior aspect of the calcaneus. This line was translated superiorly in parallel until it contacted the superior surface of the calcaneus, defining the reference line. The line was then translated further superiorly until it reached the most superior point of the posterosuperior calcaneal prominence, which was designated as the bump vertex. These measurements are illustrated in Figure 2.

Bump height was defined as the perpendicular distance between the bump vertex and the reference line. Calcaneal thickness was measured as the perpendicular distance between the bump vertex and the inferior baseline. The bump–calcaneus ratio was calculated as:

Bump–calcaneus ratio (%) = bump height/calcaneal thickness × 100

The previously reported thresholds of 4.0 mm for bump height and 7.5% for the bump–calcaneus ratio were applied [11]. For the present study, radiographic Haglund morphology was defined as the simultaneous presence of a bump height ≥ 4.0 mm and a bump–calcaneus ratio ≥ 7.5%.

The source study established 4.0 mm and 7.5% as diagnostic thresholds for the individual measurements; requiring both criteria to be satisfied was the prespecified, conservative, study-specific operational definition used in the present analysis.

### 2.5. Observer Assessment

All MRI and radiographic measurements were performed independently by two orthopaedic surgeons, each with at least three years of experience as an orthopaedic specialist. Measurements were performed on the original DICOM images using the electronic measurement tools of the PACS and were recorded at the precision displayed by the system. The observers were blinded to each other’s measurements, the patients’ clinical information, and the preassigned rupture-completeness classification. Measurements were recorded separately.

For each observer, Haglund status was derived independently from that observer’s bump height and bump–calcaneus ratio measurements. A patient was classified as having radiographic Haglund morphology when the combined threshold criteria were satisfied in both independent assessments.

For continuous analyses, the mean of the 2 observer measurements was used. Interobserver reliability was assessed separately for rupture-to-calcaneal insertion distance, bump height, and bump–calcaneus ratio.

### 2.6. Exposure, Outcome, and Covariates

The primary exposure was the presence or absence of radiographic Haglund morphology, defined by the combined criteria above; the primary outcome was rupture-to-calcaneal insertion distance.

The primary hypothesis was that Achilles tendon ruptures in patients with radiographic Haglund morphology would be located closer to the calcaneal insertion than ruptures in patients without this morphology.

Age, sex, injury mechanism, comorbidity status, rupture completeness, and treatment modality were evaluated as clinical variables. For regression analyses, injury mechanism was categorized as sport-related (football, skiing, volleyball, or basketball) or non-sport-related (twisting injury or fall).

Age, sex, sport-related injury mechanism, and the presence of any comorbidity were included in the primary adjusted analysis because they were considered clinically plausible covariates that preceded or were independent of characterization of the rupture itself. Treatment modality was not included in the adjusted model because the decision for surgical or nonoperative treatment occurred after evaluation of the rupture and could therefore represent a downstream variable.

Rupture completeness was evaluated in a sensitivity model rather than in the primary adjustment set because partial versus total rupture represents a characteristic of the rupture at presentation rather than a clearly pre-existing confounder.

### 2.7. Statistical Analysis

Continuous variables are presented as the median and interquartile range (IQR), and categorical variables as the number and percentage. Distributional characteristics of continuous variables were assessed using the Shapiro–Wilk test. The final analytic cohort contained complete data for all variables included in the statistical analyses.

Interobserver reliability for continuous imaging measurements was assessed using 2-way random-effects, absolute-agreement intraclass correlation coefficients (ICCs). Single-measure reliability was reported as ICC(2,1), and reliability of the mean of the 2 observers was reported as ICC(2,2) [20]. Ninety-five percent confidence intervals (CIs) for the ICC estimates were obtained using 10,000 nonparametric bootstrap resamples. Agreement was further evaluated using Bland–Altman analysis, including mean bias and 95% limits of agreement [21]. Agreement for the threshold-derived binary Haglund classification was assessed using Cohen κ and percentage agreement.

The primary unadjusted comparison of rupture-to-calcaneal insertion distance between patients with and without radiographic Haglund morphology was performed using a two-sided Mann–Whitney U test. The magnitude of the between-group difference was summarized using the median difference with a 95% CI derived from 20,000 bootstrap resamples and the rank-biserial correlation as an effect-size measure.

The prespecified primary adjusted analysis was performed using median regression, with rupture-to-calcaneal insertion distance as the dependent variable and radiographic Haglund morphology as the primary independent variable [22]. The model additionally included age, sex, sport-related injury mechanism, and the presence of any comorbidity. Covariates were selected on clinical grounds rather than according to univariable statistical significance.

A sensitivity median regression analysis additionally adjusted for rupture completeness. A further sensitivity analysis used ordinary least-squares regression with HC3 heteroscedasticity-robust standard errors. Potential effect modification by rupture completeness was explored by adding a radiographic Haglund morphology × rupture completeness interaction term. Post hoc sensitivity analyses applied the bump-height (≥4.0 mm) and bump–calcaneus ratio (≥7.5%) thresholds separately. Multicollinearity among model covariates was assessed using variance inflation factors.

Exploratory comparisons of categorical variables according to radiographic Haglund morphology status were performed using 2-sided Fisher exact tests, with odds ratios and 95% CIs. Age was compared using the Mann–Whitney U test. Individual injury mechanisms were summarized descriptively rather than subjected to multiple comparisons across sparse categories.

The comparison of rupture-to-calcaneal insertion distance according to radiographic Haglund morphology status represented the single confirmatory analysis. Secondary comparisons, sensitivity analyses, and interaction analyses were considered exploratory, and no adjustment for multiple testing was therefore applied.

All tests were two-sided, with statistical significance defined as *p* < 0.05. Exact *p*-values were reported unless *p* < 0.001, and effect estimates were accompanied by 95% CIs where appropriate. The study comprised all eligible patients identified during the prespecified study period; no post hoc power analysis was performed.

Statistical analyses were performed using Python version 3.13.5, primarily with SciPy version 1.17.0 and statsmodels version 0.14.6.

## 3. Results

### 3.1. Patient Selection and Characteristics

A total of 292 patients with Achilles tendon rupture were screened for eligibility. Of these, 186 were excluded: 12 were younger than 18 years, 24 had traffic accident-related injuries, 23 had an open wound and/or osteomyelitis, 14 had multitrauma, and 113 had incomplete clinical and/or imaging records. The final analytic sample comprised 106 patients (Figure 1).

The median age of the study population was 41.5 years (IQR, 37.0–51.5 years), and 27 patients (25.5%) were female. Radiographic Haglund morphology, defined by the prespecified combined criteria, was present in 28 patients (26.4%) and absent in 78 (73.6%). Patient characteristics according to radiographic Haglund morphology status are summarized in Table 1.

### 3.2. Interobserver Reliability

Interobserver reliability was excellent for all continuous imaging measurements. The ICC(2,1) was 0.9994 (95% CI, 0.9992–0.9996) for rupture-to-calcaneal insertion distance, 0.9976 (95% CI, 0.9965–0.9983) for bump height, and 0.9981 (95% CI, 0.9966–0.9989) for the bump–calcaneus ratio. Bland–Altman analyses showed minimal interobserver bias for all three measurements (Table 2 and Supplementary Figures S1–S3). The threshold-derived radiographic Haglund classification showed 100% agreement between observers (Cohen’s κ = 1.000).

### 3.3. Primary Analysis

Rupture-to-calcaneal insertion distance was shorter in patients with radiographic Haglund morphology than in those without this morphology. The median distance was 1.60 cm (IQR, 1.49–2.58 cm) in the Haglund group and 4.85 cm (IQR, 3.51–5.55 cm) in the non-Haglund group (Figure 3). The unadjusted median difference was −3.25 cm (95% CI, −3.40 to −2.68 cm; rank-biserial r = −0.696; *p* < 0.001).

### 3.4. Adjusted Analysis

In the prespecified multivariable median regression model, radiographic Haglund morphology remained associated with a shorter rupture-to-calcaneal insertion distance after adjustment for age, sex, sport-related injury mechanism, and comorbidity status (β = −2.89 cm; 95% CI, −3.61 to −2.17 cm; *p* < 0.001) (Table 3). The coefficients for the remaining model covariates are presented in Table 3.

### 3.5. Sensitivity Analyses

The association between radiographic Haglund morphology and rupture location was consistent across the prespecified sensitivity analyses (Supplementary Table S1). After additional adjustment for rupture completeness, the estimated association was −2.56 cm (95% CI, −3.27 to −1.85 cm; *p* < 0.001). The HC3 heteroscedasticity-robust ordinary least-squares model yielded an estimate in the same direction (β = −2.04 cm; 95% CI, −2.80 to −1.29 cm; *p* < 0.001). There was no evidence of effect modification by rupture completeness (radiographic Haglund morphology × total rupture interaction: β = −0.37 cm; 95% CI, −1.82 to 1.09 cm; *p* = 0.617). In the post hoc threshold analyses, the bump-height criterion identified the same 28 patients as the combined definition, whereas the bump–calcaneus ratio criterion identified 29 patients. The adjusted association remained consistent when either criterion was applied alone (bump height: β = −2.89 cm, 95% bootstrap CI, −3.37 to −1.60 cm; bump–calcaneus ratio: β = −2.88 cm, 95% bootstrap CI, −3.35 to −1.51 cm; both *p* < 0.001).

### 3.6. Exploratory Analyses

Exploratory unadjusted comparisons are summarized in Supplementary Table S2. Patients with radiographic Haglund morphology were older (median difference, 7.5 years; 95% CI, 2.0–14.5 years) and more frequently female (odds ratio, 3.11; 95% CI, 1.10–8.84). The remaining exploratory effect estimates were imprecise, with confidence intervals that included the null. These analyses were not used to select covariates for the primary model.

## 4. Discussion

The principal finding of this study was that radiographic Haglund morphology was associated with a substantially more distal location of Achilles tendon rupture. Patients with radiographic Haglund morphology had a median rupture-to-calcaneal insertion distance of 1.60 cm, compared with 4.85 cm in patients without radiographic Haglund morphology, corresponding to an unadjusted median difference of 3.25 cm. After adjustment for age, sex, sport-related injury mechanism, and comorbidity status, Radiographic Haglund morphology remained associated with a 2.89 cm shorter rupture-to-insertion distance. The association was also consistent across the prespecified sensitivity analyses. These findings support an association between posterior calcaneal morphology and the anatomical pattern of Achilles tendon rupture; they do not establish that radiographic Haglund morphology causes rupture or increases the overall risk of rupture.

The finding is relevant to sports traumatology because 60 of 106 participants (56.6%) sustained a sport-related injury. The association between radiographic Haglund morphology and rupture location persisted after adjustment for sport-related mechanism, suggesting that the observed anatomical pattern was not explained solely by whether the injury occurred during sport. Nevertheless, the study was not designed to compare specific sports or athlete populations, and the results should not be interpreted as sport-specific risk estimates.

Previous studies have shown that acute Achilles tendon ruptures are concentrated predominantly in the midsubstance region, although rupture level varies among patients. Park et al. [4] quantified rupture location on MRI in 195 patients and reported a mean distance of 6.4 cm from the distal calcaneal insertion, with most ruptures occurring 5 to 8 cm proximal to the insertion. Qureshi et al. [5] reported a mean rupture distance of approximately 5.2 cm from the enthesis and found that rupture location was clinically relevant in patients managed with functional rehabilitation. Cao et al. [6] likewise reported that rupture site may influence postoperative outcomes. In the present study, the median distance of 4.85 cm in patients without radiographic Haglund morphology was broadly consistent with the conventional midsubstance distribution. By contrast, the median distance of 1.60 cm in the morphology-positive group identified a distinctly more distal rupture pattern within this cohort.

How Haglund morphology is defined is important when interpreting this finding. Several traditional radiographic parameters, including angular measurements and parallel pitch lines, have been used to characterize posterosuperior calcaneal prominence, but their diagnostic performance and reproducibility have been inconsistent [7,8,9,23]. Tang et al. [11] proposed bump height and the bump–calcaneus ratio as quantitative radiographic measures and reported thresholds of 4.0 mm and 7.5%, respectively. We therefore used quantitative measurements rather than visual assessment alone. For this study, both thresholds were required for classification as having radiographic Haglund morphology. This combined rule should be regarded as a study-specific operational definition rather than a universally established diagnostic criterion. It is also important to distinguish radiographic Haglund morphology from symptomatic Haglund syndrome, because a prominent posterosuperior calcaneus does not necessarily imply clinical symptoms.

A plausible explanation for the observed distal shift in rupture location is the close anatomical relationship between the posterosuperior calcaneus and the distal Achilles tendon. The Achilles insertion is exposed to a combination of tensile and compressive loading that changes with ankle position and tendon excursion [13,24]. Chimenti et al. [15] demonstrated altered transverse compressive and axial tensile strain in insertional Achilles tendinopathy, supporting the concept that local mechanical conditions at the insertion differ from those in the tendon midsubstance. MRI findings of Achilles impingement have also linked posterior calcaneal morphology with localized tendon abnormalities [12]. More recently, Tang et al. [14] reported associations between quantitative Haglund measurements and Achilles tendon thickening and intratendinous degeneration in patients with insertional tendinopathy. These observations provide biological plausibility for a relationship between posterosuperior calcaneal morphology and distal tendon pathology, but evidence from tendinopathy cannot by itself be extrapolated to a causal mechanism for acute rupture.

Direct clinical evidence linking Haglund deformity with Achilles tendon rupture remains sparse. Porsch et al. [17] described an atypical rupture adjacent to a Haglund exostosis, while Madi and Hillrichs [16] reported an acute Achilles tendon rupture in the setting of Haglund deformity. A chronic rupture associated with Haglund deformity has also been reported [18]. These observations show that the conditions can coexist but, because they are isolated case reports, they cannot establish a systematic relationship between calcaneal morphology and rupture location. The present study extends this literature by examining posterior calcaneal morphology quantitatively in a clinical series and by treating rupture-to-calcaneal insertion distance as a continuous anatomical outcome.

The findings should be interpreted within the multifactorial pathophysiology of Achilles tendon rupture. Mechanical loading, intrinsic tendon degeneration, age-related changes, systemic disease, and medication exposure have all been implicated in rupture [25,26]. Histopathological studies have demonstrated degenerative abnormalities in ruptured tendons, including changes extending beyond the macroscopically apparent rupture site [27,28]. Vascular anatomy has also been investigated as a potential contributor, although the degree to which regional vascularity determines rupture location remains uncertain [24,29]. Exploratory analyses in our cohort showed that patients with radiographic Haglund morphology were older and more frequently female; sex-related differences in Achilles rupture epidemiology have been described previously [30]. However, neither age nor sex was associated with rupture distance in the primary adjusted model, and these exploratory group differences should not be interpreted as independent determinants of rupture location.

The clinical interpretation of the present findings should remain focused on rupture localization. Because all participants had already sustained an Achilles tendon rupture and no nonrupture control group was included, this study cannot determine whether radiographic Haglund morphology increases the probability of rupture. The findings instead indicate that, among patients with rupture, objectively defined Haglund morphology is associated with a more distal rupture pattern. Rupture level has been linked to clinical outcome and treatment-related considerations in previous studies [5,6]. Acute Achilles rupture management remains individualized and may be operative or nonoperative depending on patient and injury characteristics [31]. Our data do not justify changing treatment strategy, prescribing preventive interventions, performing calcaneoplasty, or considering this morphology a predictive marker on the basis of morphology alone. They support careful assessment of the distal tendon and posterior calcaneal morphology when a rupture is located unusually close to the insertion.

Several features strengthen this study. Posterior calcaneal morphology was quantified using objective radiographic measurements rather than subjective visual assessment. MRI-based rupture location was analyzed as a continuous measurement rather than being divided into arbitrary proximal and distal categories. Measurements were performed independently by 2 blinded orthopaedic surgeons, with excellent interobserver reliability for rupture distance, bump height, and the bump–calcaneus ratio and complete agreement for the threshold-derived Haglund classification. In addition, the primary association was evaluated using a prespecified adjusted model, and its direction remained stable across sensitivity analyses using alternative model specifications.

### Limitations

This study has several limitations. First, its retrospective, single-center, cross-sectional design limits temporal and causal inference. Second, 113 patients were excluded because the clinical and/or imaging records required for the study were incomplete or technically inadequate. This may have produced a selected cohort and limited representativeness. Third, chronic and neglected ruptures were excluded; therefore, the findings apply to acute ruptures and should not be extrapolated to chronic rupture patterns. Fourth, because the cohort consisted exclusively of patients with Achilles tendon rupture, the frequency of radiographic Haglund morphology could not be compared with that in individuals without rupture, and no inference regarding rupture risk can be made. Fifth, the 11-year study period may have involved variation in imaging acquisition protocols, although measurements were performed on original DICOM images using the same prespecified anatomical landmarks. Sixth, the requirement for both a bump height of at least 4.0 mm and a bump–calcaneus ratio of at least 7.5% was a study-specific operational definition. Although the individual thresholds were derived from previously published quantitative radiographic criteria [11], the combined definition requires external validation. Seventh, grouping heterogeneous conditions as “any comorbidity” may have left residual confounding, despite reporting the individual conditions by group. The retrospective dataset did not allow comprehensive adjustment for potentially relevant factors such as body mass index, smoking, detailed medication exposure, preinjury activity level, or pre-existing Achilles symptoms. Finally, preinjury imaging was unavailable, preventing assessment of the temporal sequence between posterior calcaneal morphology, distal tendon changes, and subsequent rupture.

Overall, radiographic Haglund morphology was associated with a substantially more distal location of Achilles tendon rupture, and this relationship persisted after covariate adjustment and across sensitivity analyses. Together with existing imaging and biomechanical evidence linking posterosuperior calcaneal morphology to distal Achilles tendon pathology, these findings support further investigation of the relationship between calcaneal morphology and rupture pattern. Prospective multicenter studies with standardized imaging and appropriate nonrupture comparison groups are needed to determine whether Haglund morphology contributes to the development of Achilles tendon rupture or primarily influences where rupture occurs.

## 5. Conclusions

Radiographic Haglund morphology was associated with a shorter rupture-to-calcaneal insertion distance, indicating a more distal pattern of Achilles tendon rupture. This association remained consistent after adjustment for clinically relevant covariates and across sensitivity analyses. These findings suggest that posterior calcaneal morphology may be associated with the anatomical pattern of Achilles tendon rupture; however, they do not establish a causal relationship or indicate that radiographic Haglund morphology increases the overall risk of rupture.

## Figures and Tables

**Figure 1:**
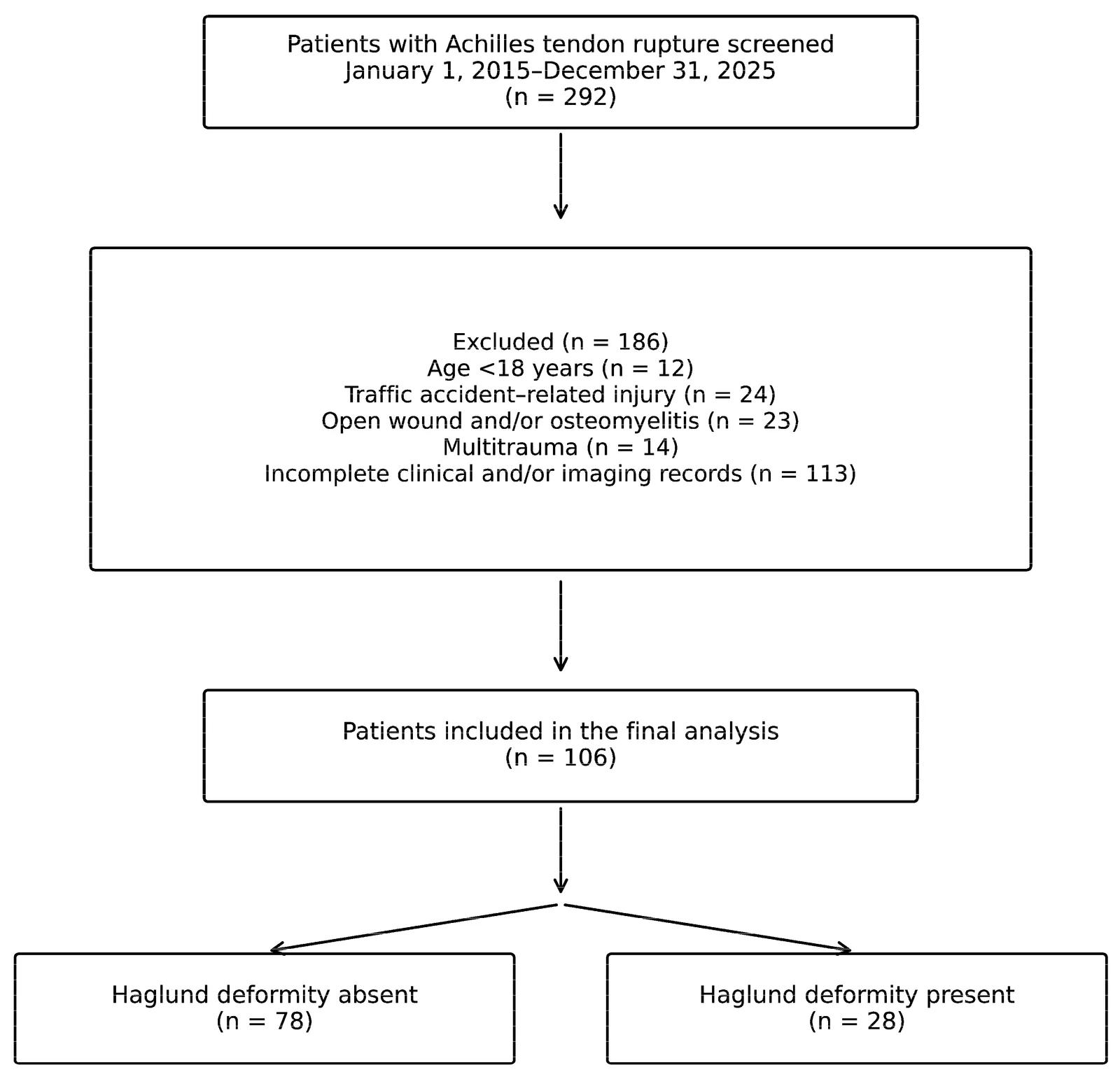
Patient selection flow diagram. A total of 292 patients with Achilles tendon rupture were screened; 186 were excluded according to the prespecified eligibility criteria, leaving 106 patients for the final analysis.

**Figure 2:**
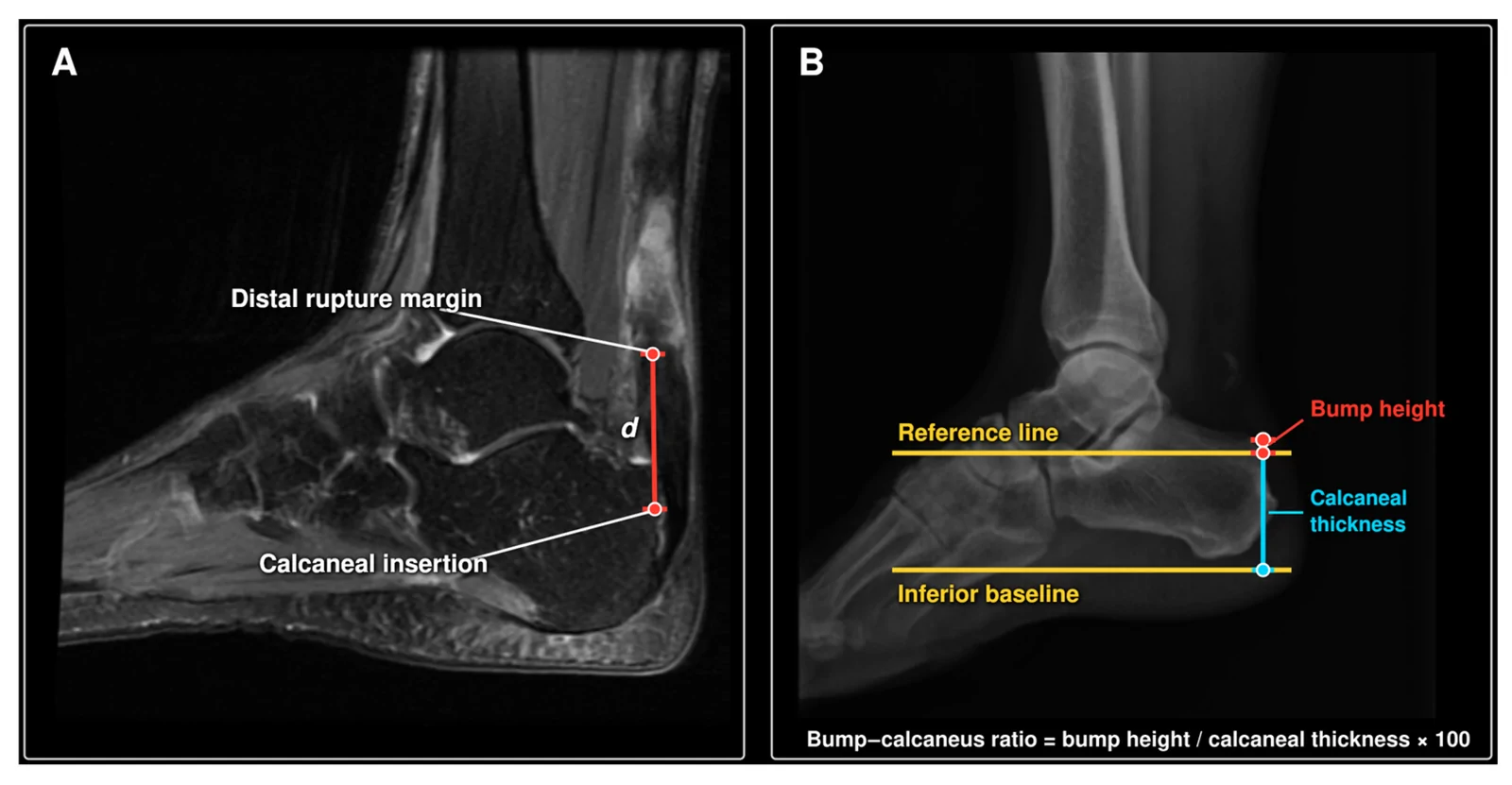
Representative MRI and lateral radiographic images illustrating the measurements. (**A**) Rupture-to-insertion distance measured on a sagittal MRI image. (**B**) Bump height and bump-to-calcaneus ratio measured on a standard lateral ankle radiograph. In panel (**A**), *d* denotes rupture-to-insertion distance.

**Figure 3:**
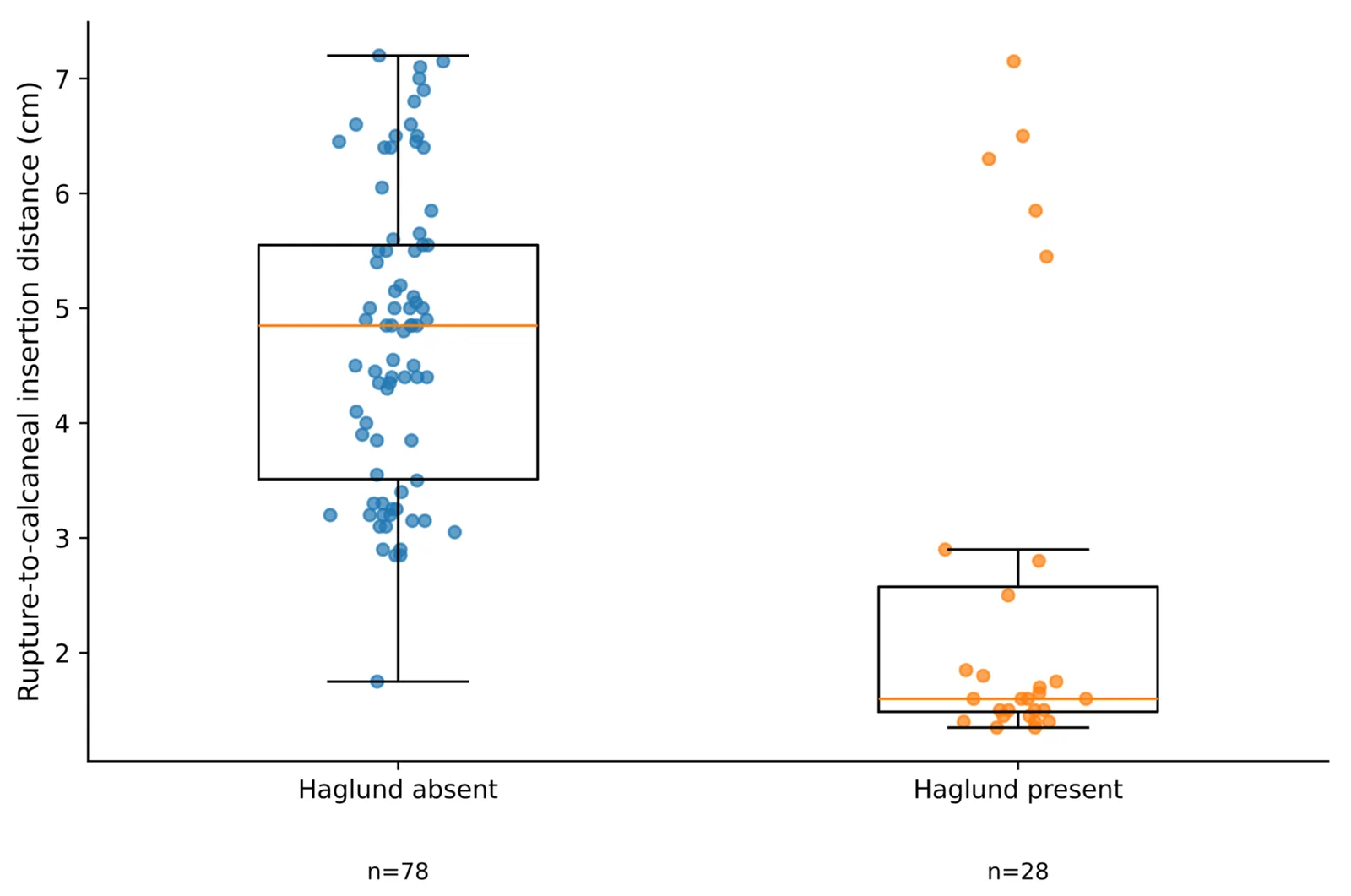
Rupture-to-insertion distance according to radiographic Haglund morphology status. Each point represents one patient. Boxes indicate the interquartile range, horizontal lines indicate the median, and whiskers extend to 1.5 times the interquartile range.

**Table 1:** Patient characteristics according to radiographic Haglund morphology status.

Variable	Overall (*n* = 106)	Haglund Present (*n* = 28)	Haglund Absent (*n* = 78)
Age, y	41.5 [37.0–51.5]	46.5 [41.8–55.8]	39.0 [36.0–48.0]
Female sex	27 (25.5%)	12 (42.9%)	15 (19.2%)
Right side	51 (48.1%)	13 (46.4%)	38 (48.7%)
Sport-related mechanism	60 (56.6%)	11 (39.3%)	49 (62.8%)
Total rupture	48 (45.3%)	16 (57.1%)	32 (41.0%)
Any comorbidity	36 (34.0%)	14 (50.0%)	22 (28.2%)
Diabetes mellitus	21 (19.8%)	8 (28.6%)	13 (16.7%)
Chronic kidney disease	14 (13.2%)	5 (17.9%)	9 (11.5%)
Hypertension	5 (4.7%)	2 (7.1%)	3 (3.8%)
Osteoporosis	3 (2.8%)	3 (10.7%)	0 (0.0%)
Gout	1 (0.9%)	0 (0.0%)	1 (1.3%)
Surgical treatment	19 (17.9%)	4 (14.3%)	15 (19.2%)
Football	37 (34.9%)	9 (32.1%)	28 (35.9%)
Twisting injury	30 (28.3%)	12 (42.9%)	18 (23.1%)
Fall	16 (15.1%)	5 (17.9%)	11 (14.1%)
Skiing	10 (9.4%)	1 (3.6%)	9 (11.5%)
Volleyball	7 (6.6%)	1 (3.6%)	6 (7.7%)
Basketball	6 (5.7%)	0 (0.0%)	6 (7.7%)

Values are median [IQR] or *n* (%). IQR, interquartile range; y, years. Comorbidity categories were not mutually exclusive. Inferential comparisons of patient characteristics were exploratory and are provided in Supplementary Table S2.

**Table 2:** Interobserver reliability and agreement of imaging measurements.

Measurement	ICC(2,1) (95% CI)	ICC(2,2) (95% CI)	Bias (R1–R2)	95% Limits of Agreement
Rupture-to-insertion distance, cm	0.9994 (0.9992–0.9996)	0.9997 (0.9996–0.9998)	−0.006 cm	−0.125 to 0.113 cm
Bump height, mm	0.9976 (0.9965–0.9983)	0.9988 (0.9982–0.9992)	−0.0005 mm	−0.107 to 0.106 mm
Bump–calcaneal ratio, %	0.9981 (0.9966–0.9989)	0.9990 (0.9983–0.9995)	−0.0066%	−0.148% to 0.135%
Haglund classification	100%; κ = 1.000	—	—	—

ICC(2,1), two-way random-effects, absolute-agreement, single-measure intraclass correlation coefficient; ICC(2,2), reliability of the mean of two observers; CI, confidence interval. The 95% CIs for the ICCs were obtained from 10,000 bootstrap resamples. Bias and 95% limits of agreement were derived from Bland–Altman analyses. Agreement for the threshold-derived radiographic Haglund classification was summarized using percentage agreement and Cohen’s κ.

**Table 3:** Prespecified multivariable median regression for rupture-to-calcaneal insertion distance.

Predictor	β, cm	95% CI	*p* Value
Haglund present	−2.89	−3.61 to −2.17	<0.001
Age, per year	−0.04	−0.08 to 0.01	0.100
Female sex	0.28	−0.50 to 1.06	0.476
Sport-related mechanism	0.60	−0.31 to 1.50	0.195
Any comorbidity	0.84	−0.26 to 1.93	0.132

Negative β values indicate a rupture location closer to the calcaneal insertion. CI, confidence interval. Covariates were selected a priori on clinical grounds and not according to univariable *p*-values.

## Data Availability

The datasets generated and/or analyzed during the current study are not publicly available because they contain potentially identifiable clinical and imaging information. De-identified data may be made available from the corresponding author upon reasonable request, subject to ethics committee and institutional approval and applicable patient-confidentiality restrictions.

## Supplementary Materials

The following supporting information can be downloaded at: https://www.mdpi.com/article/10.3390/diagnostics16213592/s1, Table S1, sensitivity analyses for rupture-to-calcaneal insertion distance; Table S2, exploratory comparisons according to radiographic Haglund morphology status; Figure S1, Bland–Altman plot of interobserver agreement for rupture-to-calcaneal insertion distance; Figure S2, Bland–Altman plot of interobserver agreement for bump height; Figure S3, Bland–Altman plot of interobserver agreement for the bump–calcaneus ratio.

## Abbreviations

CI, confidence interval; DICOM, Digital Imaging and Communications in Medicine; HC3, heteroscedasticity-consistent covariance estimator type 3; ICC, intraclass correlation coefficient; IQR, interquartile range; MRI, magnetic resonance imaging; OLS, ordinary least squares; STROBE, Strengthening the Reporting of Observational Studies in Epidemiology.
