# Supplementary material for: Association Between Radiographic Haglund Morphology and MRI-Measured Achilles Tendon Rupture Location: A Retrospective Cross-Sectional Study
Source: Diagnostics (Basel). 2026 Oct 30;16(21):3592. doi: 10.3390/diagnostics16213592 (PMC13761418; doi:10.3390/diagnostics16213592)
Supplement: Supplementary file 1 [file diagnostics-16-03592-s001.zip › diagnostics-4603181-supplementary.pdf]

# Supplementary Materials

## Association between Radiographic Haglund Morphology and MRI-Measured Achilles Tendon Rupture Location: A Retrospective Cross-Sectional Study

**Table S1.** Sensitivity Analyses for Rupture-to-Calcaneal Insertion Distance

| Analysis | Definition/Predictor | β, cm | 95% CI | P value |
|---|---|---|---|---|
| Median regression + rupture completeness | Haglund present | −2.56 | −3.27 to −1.85 | <0.001 |
| OLS with HC3 robust SE | Haglund present | −2.04 | −2.80 to −1.29 | <0.001 |
| Interaction median regression | Haglund × total rupture | −0.37 | −1.82 to 1.09 | 0.617 |
| Separate-threshold median regression | Bump height ≥4.0 mm | −2.89 | −3.37 to −1.60 | <0.001 |
| Separate-threshold median regression | Bump-calcaneus ratio ≥7.5% | −2.88 | −3.35 to −1.51 | <0.001 |

*CI, confidence interval; HC3, heteroscedasticity-consistent covariance estimator type 3; OLS, ordinary least squares; SE, standard error. Separate-threshold analyses were post hoc and used 10,000 bootstrap resamples for the reported CIs.*

**Table S2.** Exploratory Comparisons According to Radiographic Haglund Morphology Status

| Variable | Haglund present | Haglund absent | Effect estimate (95% CI) |
|---|---|---|---|
| Age, y | 46.5 [41.8–55.8] | 39.0 [36.0–48.0] | Median difference 7.5 y (2.0 to 14.5) |
| Female sex | 12 (42.9%) | 15 (19.2%) | OR 3.11 (1.10 to 8.84) |
| Right side | 13 (46.4%) | 38 (48.7%) | OR 0.91 (0.35 to 2.37) |
| Sport-related mechanism | 11 (39.3%) | 49 (62.8%) | OR 0.39 (0.14 to 1.01) |
| Total rupture | 16 (57.1%) | 32 (41.0%) | OR 1.90 (0.73 to 5.07) |
| Any comorbidity | 14 (50.0%) | 22 (28.2%) | OR 2.52 (0.95 to 6.79) |
| Surgical treatment | 4 (14.3%) | 15 (19.2%) | OR 0.70 (0.15 to 2.51) |

*Values are median [IQR] or n (%). CI, confidence interval; IQR, interquartile range; OR, odds ratio; y, years. These analyses were exploratory and were not used for covariate selection in the primary model.*

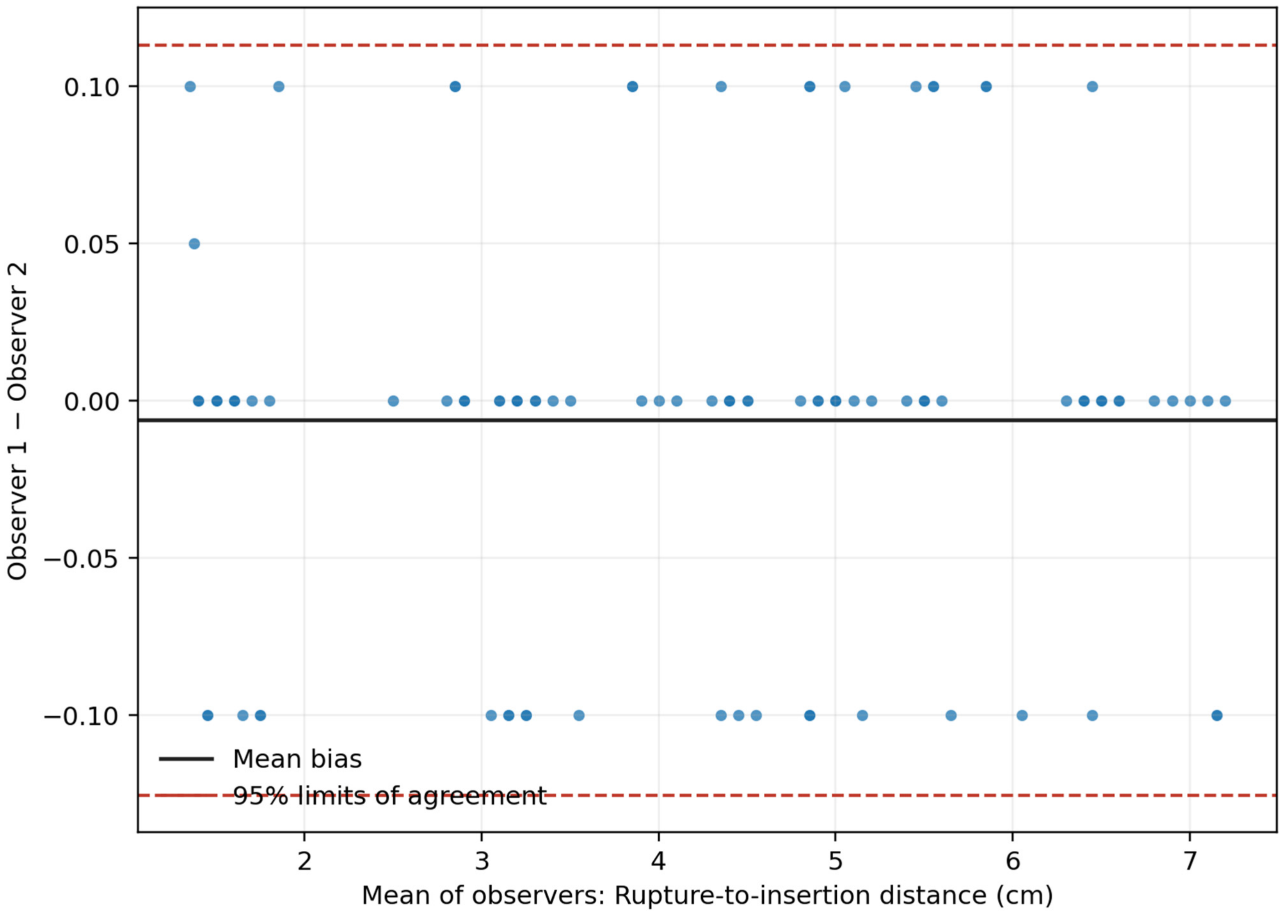


**Figure S1.** Bland–Altman plot of interobserver agreement for rupture-to-calcaneal insertion distance.

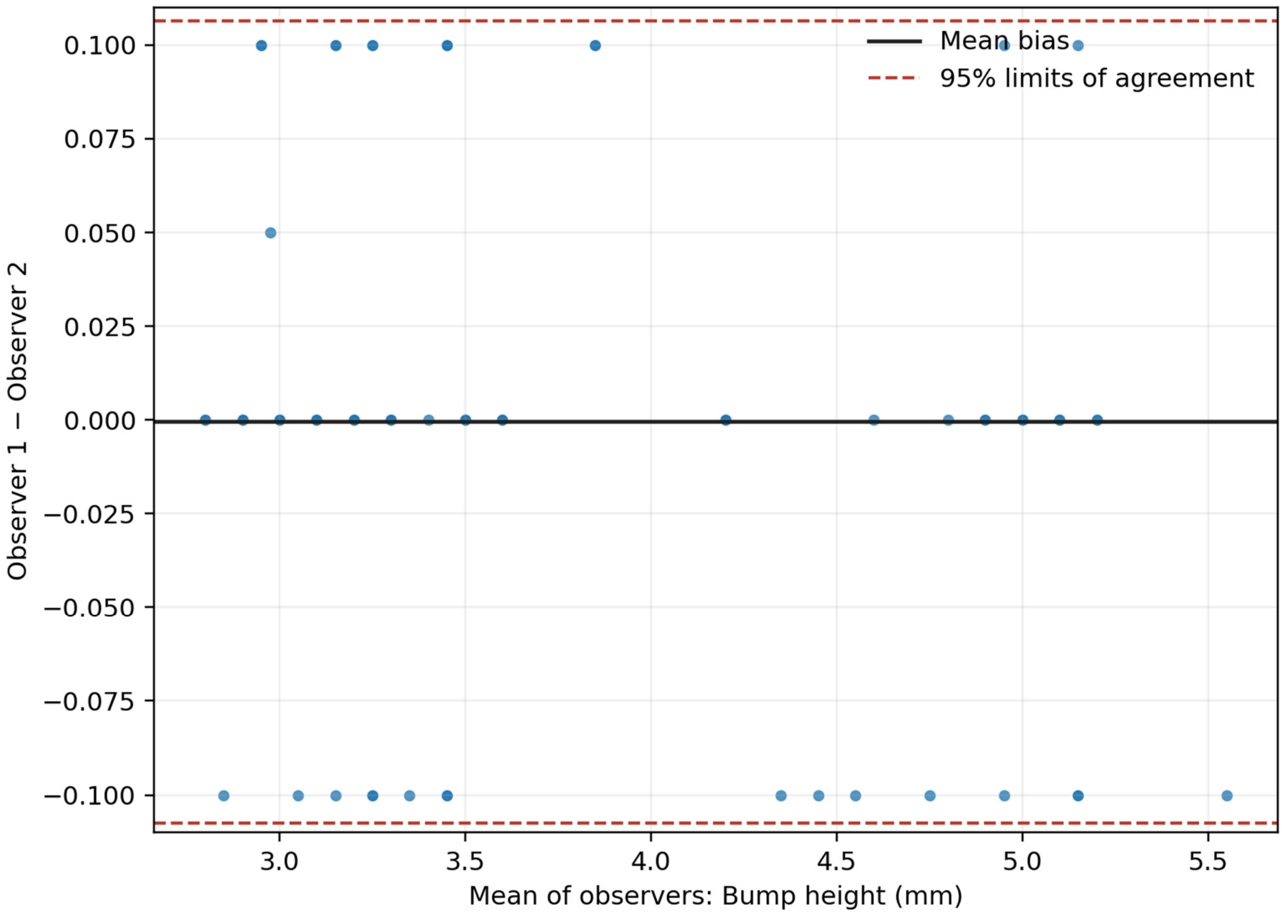


**Figure S2.** Bland–Altman plot of interobserver agreement for bump height.

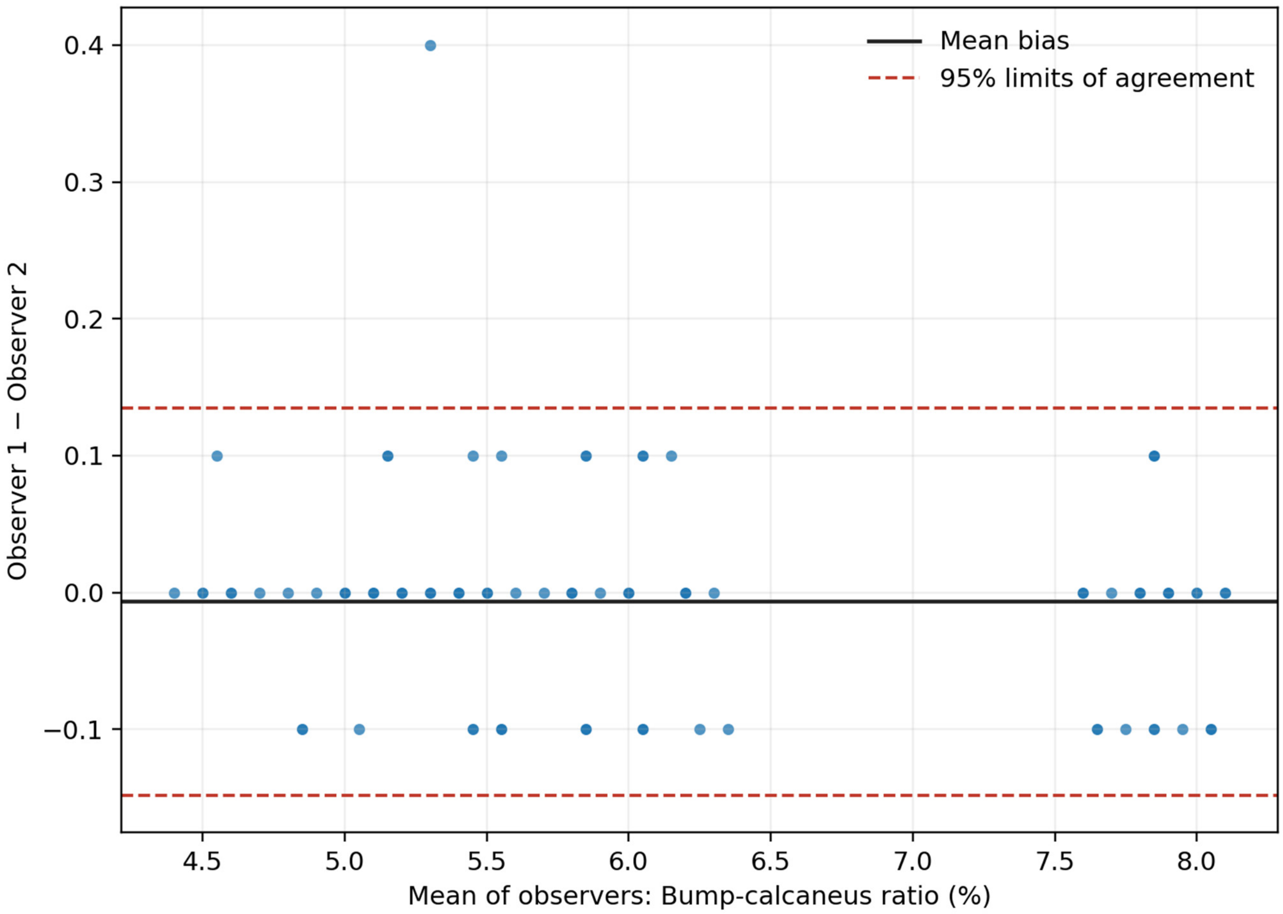


**Figure S3.** Bland–Altman plot of interobserver agreement for the bump-calcaneus ratio.
